# THE INFLUENCE OF EMOTIONAL IQ ON THE LAGGED RELATIONSHIPS BETWEEN MOMENTARY PAIN AND AFFECT IN OLDER ADULTS

**DOI:** 10.1093/geroni/igz038.1132

**Published:** 2019-11-08

**Authors:** Shelley E Condon, Brian Cox, Dylan M Smith, Patricia A Parmelee

**Affiliations:** 1 The University of Alabama, Tuscaloosa, Alabama, United States; 2 Alabama Research Institute of Aging, Tuscaloosa, Alabama, United States; 3 Stony Brook University, Stony Brook, New York, United States

## Abstract

Emotion regulation is influenced by stage of life and time perspective (Carstensen, 1992), with older adults placing greater emphasis on optimizing positive moods and repairing negative ones. Recently, there has been growing interest in emotional intelligence (EI) as a broad indicator of these mood regulation processes. Multiple cross-sectional studies have examined EI, pain and affect in older adults with chronic pain; however, little research has addressed these relationships in temporal context. The current microlongitudinal study addressed this gap by examining (1) lagged relationships between momentary pain and affect, (2) main effects of emotional intelligence (mood attention, clarity and repair) on those relations, and (3) the moderating role of EI on lagged relationships between pain and affect. Three hundred twenty-five older adults (mean age = 63.9) with knee osteoarthritis completed in-person interviews and received four phone calls daily (random within 4-hour blocks) for one week. Multilevel models examined the predictive value of affect from the previous call on current pain, and vice versa, controlling for sociodemographic variables. Across all outcomes (positive affect, negative affect, pain), a significant main effect was found for mood clarity and repair, but not attention. However, EI did not moderate lagged associations between momentary pain and affect. Average pain (across the 28 calls) significantly predicted momentary negative affect, and vice versa. Thus, while emotional intelligence is significantly related to momentary mood states, it does not appear to be related to momentary pain. Implications and ideas for future research are discussed. (R01-AG041655, P. Parmelee & D. Smith, Co-PIs)

